# Effect of the Aqueous Quercetin Solution on the Physiological Properties of Virginia Mallow (*Ripariosida hermaphrodita*) Grown Under Salt Stress Conditions

**DOI:** 10.3390/ijms26031233

**Published:** 2025-01-30

**Authors:** Dagmara Migut, Michał Sobaszek, Marta Jańczak-Pieniążek, Karol Skrobacz

**Affiliations:** 1 Department of Crop Production, University of Rzeszów, Zelwerowicza 4, 35-601 Rzeszów, Polandmjanczak@ur.edu.pl (M.J.-P.); 2 Department of Soil Science, Environmental Chemistry and Hydrology, University of Rzeszów, Zelwerowicza 8B, 35-601 Rzeszów, Poland; kskrobacz@ur.edu.pl

**Keywords:** energy plants, abiotic stresses, gas exchange, chlorophyll content, chlorophyll fluorescence

## Abstract

The current increase in energy demand, along with the deepening climate crisis, has led to the need for alternative energy sources. One of these is the cultivation of energy crops. In turn, issues related to the deepening problem of soil salinization are an important aspect of environmental protection on a global scale. New species and innovative solutions are sought to support the effective cultivation of energy crops, including in saline areas. The purpose of the study was to evaluate the effect of the foliar application of an aqueous quercetin solution applied in different doses (1%, 3% and 5%) on the physiological properties of Virginia Mallow plants subjected to salt stress conditions. The experiment was carried out as a pot experiment. The results obtained were related to two types of plants treated as a control sample. In one case, they were grown with the addition of quercetin alone, without salt stress. The other group was grown without quercetin and without salt. Quercetin is a phenolic compound that plays an important physiological and biochemical role in plants. Salinity caused a significant decrease in physiological indices in Virginia Mallow leaves. Foliar application of an aqueous quercetin solution mitigated the negative impact of salt on plants, the most stimulating effect being demonstrated at a dose of 5.0%.

## 1. Introduction

Since the industrial revolution in Europe, the consumption of fossil fuels has increased along with the growth of the population and the improvement in living standards, and the dynamic development of national economies requires a significant increase in energy demand [1]. Conventional energy resources, such as coal, natural gas, and crude oil, are no longer sufficient to meet the energy needs of the world economy, and the rapid increase in energy intensity in many industries may result in the depletion of these resources [2]. For each national economy, achieving energy security is a primary goal [3,4], both in terms of energy efficiency and environmental sustainability [5,6]. Energy crops are annual or perennial fast-growing species, characterized by the production of a large amount of biomass per unit area. They are mainly intended for the production of biogas or direct combustion [7,8]. When deciding to grow crops for energy purposes, it is necessary to take into account local climate and soil conditions, as well as the desired characteristics of the species: high dry matter yield, high calorific value, low energy input, and low nutrient demand [9].

Virginia Mallow (*Ripariosida hermaphrodita* (L.) Weakley and D.B. Poind; synonym *Sida hermaphrodita* (L.) Rusby) is a perennial crop plant originating in the southeast regions of North America, without special soil or climatic requirements [10]. Virginia Mallow was originally introduced to the former USSR in the 1930s as a feedstock and then introduced to Poland in the 1950s [11]. It can be grown for up to 20–30 years and achieve high annual biomass yields of 20–25 t∙ha^−1^ [12]. The cultivation of this plant is characterized by a wide potential for use, that is, as an energy plant, a food plant and for industrial and medicinal use [13]. In addition, Virginia Mallow plantations have a positive effect on soil quality [13,14,15,16]. It is a plant with a long flowering time, and its flowers are a source of nectar and pollen for honeybees [12] and other pollinating insects, which is a very important aspect of environmental protection. Therefore, Virginia Mallow has great potential in combining ecosystem services, such as reducing soil erosion [13], carbon storing [15], phytoremediation [16], and leaching of nitrates, as well as enhancing biodiversity [14]. In recent decades, Virginia Mallow has been cultivated mainly as an energy crop. Recent studies on Virginia Mallow have shown that its biomass is useful as a solid fuel, with the advantage of good processability (harvesting, pelleting, briquetting), good combustion properties, high ash melting point, and a generally good comprehensive life cycle assessment [17,18,19]. The most promising application of Virginia Mallow appears to be in the production of biogas for energy purposes [13].

However, in any agricultural production, plants are exposed to various types of environmental stress. Salt stress negatively affects germination, plant growth, nutrient uptake, and yield [20,21,22]. It causes oxidative stress in plants and changes in the activity of antioxidant enzymes [23,24]. Plants have developed a complex network of interconnected systems to prevent species extinction. These systems operate through a cascade of molecular events that allow for rapid responses and adaptations to adverse conditions [25]. Stress responses include the perception of signals, their transmission, and the expression of stress response genes [26]. First, plant cells detect stress using sensors or receptors located in the cell wall or plasma membrane [27]. Second transmitters, such as reactive oxygen species (ROS), calcium ions, and lipid-derived signalling molecules, transform basic external signals into intracellular signals [28]. Phytohormones, including auxin, cytokinin, gibberellic acid, abscisic acid (ABA), jasmonic acid (JA), ethylene, and salicylic acid, also play an important role as second key mediators that help coordinate signalling pathways in response to stress [29]. Several transcription factors (TFs) closely related to different types of stress, such as WRKY, MYB, AP2/ERF, and bZIP, have been identified in the literature. In response to external signals, plants synthesise, express, and perform the function of signalling and activating the expression of subsequent genes, allowing them to better adapt to changing environmental conditions [30]. The WRKY transcription factor plays a key role in physiological processes, such as ageing and development, and plant responses to abiotic stimuli, such as salinity, drought, temperature changes, and other environmental variables. The importance of WRKY lies in its key role in various signalling cascades and regulatory networks that influence plant defence responses. This factor regulates gene expression through complex interactions and self-regulatory feedback loops [31]. Salinity limits crop growth by affecting many processes that are dependent or independent of salt accumulation in shoots [22,24,32]. Depending on the intensity and duration of salt exposure, shoot biomass is further reduced as a result of premature leaf senescence and of salt accumulation in leaves at toxic levels. Initial plant responses to salt stress generally consist of reduced leaf expansion and partial/complete stomatal closure [33]. These responses are conditioned by increased accumulation of stress hormones, especially ABA. In addition, yield reduction under stress conditions is associated with disturbances in carbon metabolism and transport [34]. Salts can inhibit plant growth through primary and secondary damage. Primary salt damage refers to direct damage by salt ions [35]. A large number of Na^+^ and Cl^−^ ions in the root environment of plants can cause osmotic stress and ion toxicity [36]. Excessive accumulation of ions can lead to the degeneration and inactivation of some enzymes in plant cells, affecting normal physiological functions and metabolic activity [37]. Salt stress can also cause the accumulation of ROS in plants, which can further damage the cell membrane [38]. Secondary damage caused by salt stress includes osmotic, water and nutrient stress in plants caused by the indirect effects of salt ions. Excess soil salinity reduces the osmotic potential of soil solution in the root environment, resulting in reduced water absorption by plant roots and in physiological drought [39].

Various chemical compounds are increasingly used for protection and stimulation in plant production. New substances are being sought that positively affect plant growth and development and increase tolerance to various biotic and abiotic stresses, which also translate into higher yields. Quercetin (3,3′,4′,5,7-pentahydroxyflavone), classified as a flavanol, is one of six subclasses of flavonoid compounds [40]. Flavonoids are a diverse group of secondary metabolites that perform a wide range of biological functions, including protection against stress [41]. Increased flavanol content under the influence of biotic and abiotic stress indicates its stress-filtering function in plants. Quercetin facilitates several physiological processes in plants, such as germination, growth, antioxidant mechanisms, and photosynthesis, and also improves proper plant development. Furthermore, it is a strong antioxidant, so it provides tolerance to biotic and abiotic stresses [40]. The antioxidant properties of quercetin make it a more reliable source of stress control, since most stresses harm the plant by generating oxidative stress (via ROS production). Quercetin-mediated stress relief suggests that quercetin has a protective function [42]. Unfortunately, only a few experiments have been conducted to demonstrate its ability to alleviate stress [43,44,45].

It is estimated that intensive biomass production can negatively affect food production due to insufficient agricultural land [46]. Therefore, marginal land, where the cultivation of species for food purposes is impossible, limited or inefficient, e.g., due to high salinity, can be used for energy crop plantations. The use of substances that support plant growth under stress conditions could have a positive impact on supporting energy crops in degraded areas and their more efficient production and thus contribute to increased biomass production and support for energy production. One such substance is quercetin, an aqueous solution of which affects the physiological properties of energy crops. In addition, Virginia mallow is among the example plants, allowing the potential development of areas with increased salt content in the soil. The purpose of the experiment was to see how and to what extent the application of an aqueous solution of quercetin would compensate for salt stress affecting plant growth caused by the addition of sodium chloride to the soil in a pot experiment.

## 2. Results

### 2.1. Gas Exchange

**Figure 1:**
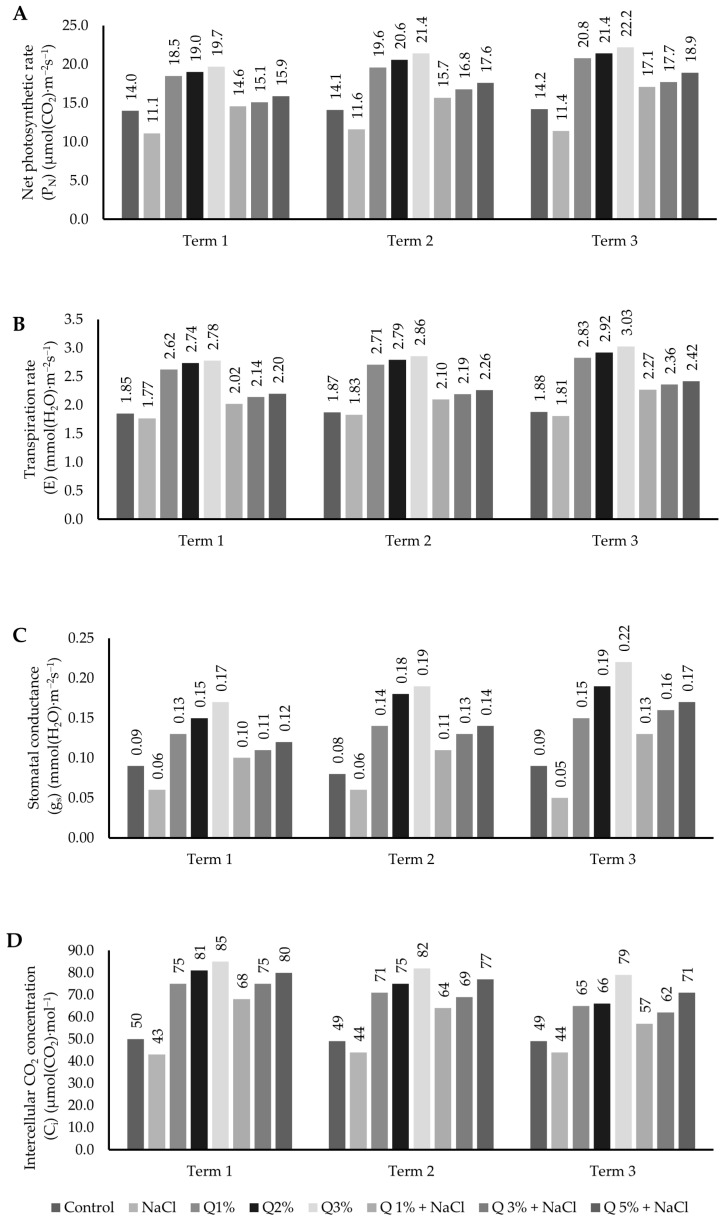
Effect of concentrations of aqueous quercetin solutions on: (**A**) net photosynthetic rate (P_N_), (**B**) transpiration rate (E), (**C**) stomatal conductance (g_s_), (**D**) intercellular CO_2_ concentration (C_i_) in leaves of Virginia Mallow grown under salt stress conditions. Capital letters represent significant differences between means in subsequent measurement dates; lower case letters indicate differences between variants at a particular measurement date (*p* ≤ 0.05).

In the studies conducted, a significant (*p* ≤ 0.05) effect of the applied concentration of quercetin was observed on gas exchange parameters in the leaves of Virginia Mallow grown under salt stress conditions (Figure 1). Similarly, an increase in the parameters analysed was observed in plants in which salt stress was not applied. Both the dose and the measurement date significantly conditioned the values of the parameters analysed. The values of the parameters net photosynthetic rate (P_N_), transpiration rate (E), and stomatal conductance (g_s_) after the application of all concentrations differed significantly in comparison with the control at each of the measurement dates analysed. In the case of intercellular CO_2_ concentration (C_i_), a decrease in the value of the parameter analysed was observed during the experiment, regardless of the concentration of quercetin applied. Compared to the control, the most stimulating effect of quercetin on gas exchange parameters (P_N_, E, g_s_, C_i_) in Virginia Mallow plants grown under saline conditions was noted at the third measurement date. However, this relationship was visible throughout the experiment. The highest concentration (5%) had the most beneficial effect on the gas exchange parameters.

### 2.2. Chlorophyll Content Index

**Figure 2:**
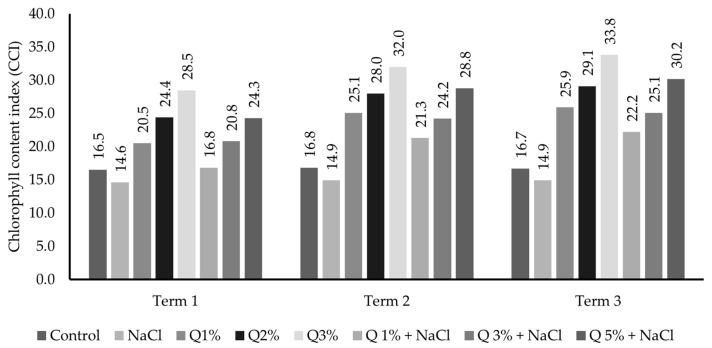
The effect of quercetin aqueous solution concentrations on chlorophyll content index (CCI) in Virginia Mallow leaves grown under salt stress conditions. Capital letters represent significant differences between means in subsequent measurement dates; lower case letters indicate differences between variants at a particular measurement date (*p* ≤ 0.05).

In the studies conducted, the use of an aqueous solution of quercetin had a positive effect on the increase in the chlorophyll content index (CCI) in the leaves of Virginia Mallow (Figure 2). Regardless of the concentration used, these values were statistically significant (*p* ≤ 0.05) compared to the control. The highest concentration (5%) had the most beneficial effect on the CCI. After lower concentrations were used, an increase in the value of the analysed parameter was also observed, but this was not as dynamic. Furthermore, for the measurement dates, the increase in quercetin concentration caused an increase in the relative chlorophyll content in the leaves of the analysed plant.

### 2.3. Chlorophyll Fluorescence

**Figure 3:**
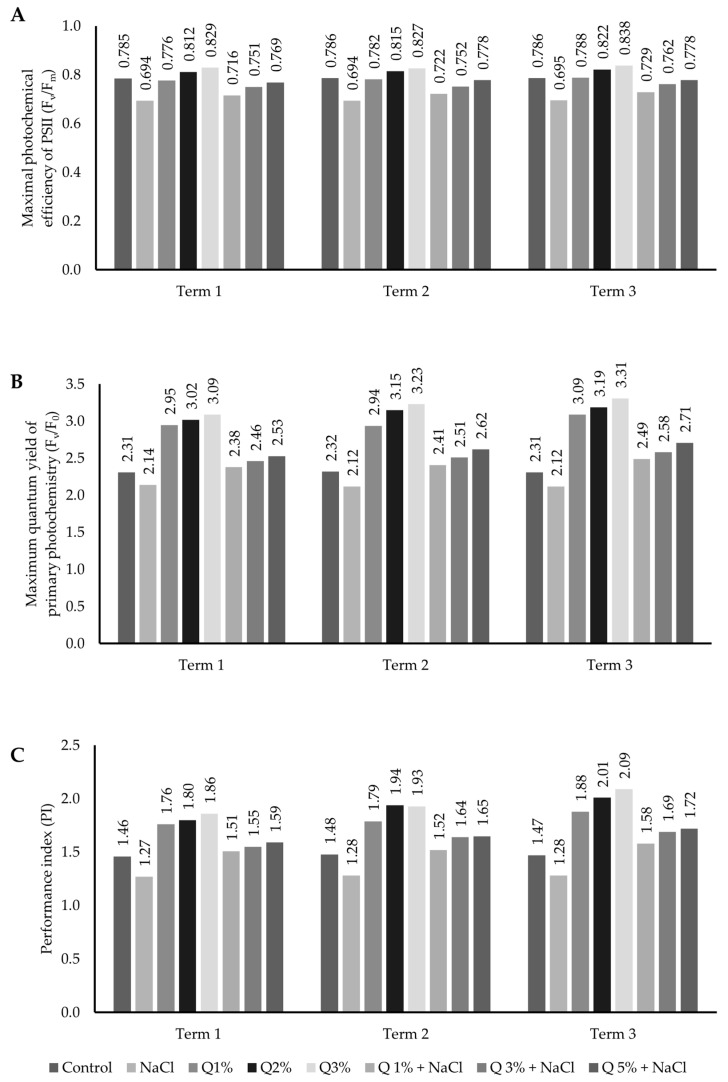
Effect of concentrations of aqueous quercetin solutions on (**A**) the photochemical efficiency of PS II (F_v_/F_m_); (**B**) the maximum quantum yield of primary photochemistry (F_v_/F_0_); and (**C**) the performance index of PS II (PI) in leaves of Virginia Mallow grown under salt stress conditions. Capital letters represent significant differences between means at subsequent measurement dates; lower case letters indicate differences between variants at a particular measurement date (*p* ≤ 0.05).

Chlorophyll fluorescence values expressed as maximal photochemical efficiency of PSII (F_v_/F_m_), maximum quantum yield of primary photochemistry (F_v_/F_0_), and performance index (PI) were significantly (*p* ≤ 0.05) conditioned by the dose of the applied solution and the measurement date (Figure 3). The foliar application of the aqueous quercetin solution stimulated the chlorophyll fluorescence parameters in Virginia Mallow plants. The maximum efficiency of the water decomposition reaction on the PSII side (F_v_/F_m_) depended on the applied quercetin concentration and the duration of the experiment. A stimulating effect was found for all concentrations analysed on each measurement date. A similar relationship was observed for F_v_/F_0_ and PI. In the experiment conducted, a significant difference was observed in PI values with respect to the applied quercetin dose. The values of the analysed parameters increased with the increase in quercetin concentration, as well as with the duration of the experiment. The values of the analysed parameters increased with the increase in quercetin doses compared to the control. It appears that the highest concentration of quercetin solution used (5%) had the most stimulating effect on chlorophyll fluorescence parameters.

## 3. Discussion

Currently, the growing environmental pollution and climate change caused by conventional energy sources are a global problem [47,48,49]. The depletion of fossil fuel resources forces the search for more innovative energy sources, which is why renewable energy sources, including biomass, are becoming increasingly popular. Many organizations, such as the European Union (EU), have begun implementing activities to protect the natural environment and promote alternative solutions regarding nonconventional energy sources. Modern society is more aware of growing environmental problems on a global scale. This leads to an increase in interest in renewable energy [50]. Compared to traditional fossil fuels, biomass is widely available and has a lower impact on the environment. In addition, the cultivation of energy crops contributes to the reduction of carbon dioxide. However, any environmental stress, including soil spread and water salinization, has a destructive effect on plant production [51,52]. Salt stress adversely affects soil properties, plant growth, and overall productivity [53,54]. One of the early symptoms of salt stress in plant tissue is a decrease in relative water content (RWC). Reduction in RWC in stressed plants may be associated with a decrease in plant vigor and has been observed in many species [55]. Cetner et al. [56] report that the productivity of Virginia mallow is sensitive to unfavorable physicochemical properties of the soil, and temporary water deficiency may cause a significant decrease in productivity, and thus a reduction in yields. Another response to abiotic stress may be closure of the stomata in plants [57]. Unfavourable environmental conditions allow them to increase their respiration rate. This is a necessary condition for the production of ATP in order to activate osmotically soluble substances (activation of cells under stress), which reduce the osmotic potential of the cell, thus increasing its water uptake [58]. Therefore, new methods are sought to reduce the effects of stress and prevent its occurrence.

Antioxidants, including quercetin, belong to a group of organic compounds that, through osmotic regulation, can play an important role in alleviating stress associated with environmental factors, such as drought, high temperature, and salinity, and thus positively affect the course of gas exchange [59,60]. Quercetin, which is a strong antioxidant, increases tolerance to biotic and abiotic stresses in plants [61,62]. In studies carried out on the effect of exogenous application of an aqueous solution of quercetin on plants of Virginia Mallow, a stimulating effect of this compound was found on the course of physiological processes. Other authors also indicated that the use of quercetin can change the physiological properties of plants. Dobrikovea and Apostolova [63] showed that the use of quercetin can change photosynthetic properties caused by structural changes in thylakoid membranes, which can have an effect on increasing the transfer from PSII to PSI, improving the photosynthesis process. Yildiztugay et al. [64] showed a positive effect of flavonoids on the photosynthesis process in common beans. In our studies, after the use of the flavonoid quercetin, an increase in the values of chlorophyll fluorescence parameters and gas exchange was found in comparison to the control, which contributed to the improvement in the photosynthesis process. As a result of spraying, a positive effect of quercetin was demonstrated on the value of the physiological parameters studied (chlorophyll content, values of selected chlorophyll fluorescence parameters, and gas exchange), proportional to the concentrations used. In our own studies, an increase in the values of the gas exchange parameters (P_N_, E, g_s_) was observed as a result of the application of quercetin, as well as a positive effect of quercetin on the value of the intercellular CO_2_ concentration (C_i_). The strongest response of the plants to the foliar application of quercetin appears to be observed on the third measurement date. In the first and second dates, an increase in the analysed parameters is observed; however, this is not so dynamic. Environmental stresses limit the photosynthesis process mainly by reducing stomatal conductance, causing oxidative stress. As a result, the activity of ribulose-1,5-biphosphate carboxylase/oxygenase (RUBISCO), which is responsible for the fixation of carbon dioxide in the dark phase of photosynthesis, decreases [65]. The reduction in RUBISCO content in maize leaves is one of the reasons for the reduced photosynthetic capacity of plants subjected to stress and may contribute to toxic premature aging and reduce the CO_2_ fixation process in the Calvin–Benson cycle [66]. The increase in gs, found as a result of the action of a quercetin derivative, reduced the accumulation of intracellular CO_2_ in the mesophyll and caused a decrease in the C_i_ value. This phenomenon was accompanied by an increase in the intensity of P_N_. In studies by other authors, the value of CO_2_ concentration during gas exchange for Virginia Mallow is on average between 5.8 and 9.9 μmol∙m^−2^s^−1^ [67]. Other studies showed higher concentrations of 12–17 μmol∙m^−2^s^−1^ [68].

Transcription factors play a key role in reducing abiotic stresses, including salt stress. Han et al. [69] were able to isolate a new gene from *Malus xiaojinensis*. The overexpression of the *MxWRKY64* gene in transgenic Arabidopsis thaliana contributed to increased tolerance to salt stress. Jun et al. [70] cloned and characterised the *MbERF12* gene derived from *Malus baccata*. Its overexpression in *Arabidopsis thaliana* significantly enhanced tolerance to abiotic stress through ethylene signal transduction and also enhanced the antioxidant enzyme system and maintained ROS homeostasis and membrane lipid stability under stress conditions. Salt stress also affected the expression of the *MxWRKY53* gene obtained from *Malus xiaojinensis* [71]. The transcription factor *TaWRKY31*, a member of the wheat WRKY II family, enhances tolerance to drought in *Arabidopsis thaliana* by helping to eliminate ROS, reducing stomatal aperture, and increasing the expression of stress-responsive genes [72]. The WRKY gene family has also been studied in *Rosa chinensis*, revealing that the expression of the *RcWRKY* gene is tissue specific and increased levels are observed under conditions of drought, high temperature and salt stress [73]. In *Arabidopsis thaliana*, it was confirmed that the *MbWRKY53* genes can improve plant tolerance to cold and drought stress through various pathways, including the CBF pathway, the SOS pathway, the proline synthesis pathway and ABA-dependent pathways [74]. WRKY transcription factors play an important role in the regulation of transcriptional reprogramming, which is associated with plant responses to biotic and abiotic stresses [75]. Salt stress causes chloroplast damage and yellowing of plants, so chlorophyll content is one of the key indicators that allow assessing whether a plant is experiencing adverse stress. The WRKY factor is critical for plant responses to environmental stressors because it selectively inhibits non-essential gene pathways [76]. Pigment compounds, including chlorophylls and carotenoids, are important phytochemicals that are not only essential for the proper development of plant physiological functions, but also stand out as powerful antioxidants with numerous beneficial biological functions and health benefits [77,78,79]. In the early stages of plant development, the chlorophyll content in the leaves increases and then, with the aging process, it decreases and other pigments accumulate. Chlorophyll is one of the most important biochemical features related to water availability and the level of plant nutrition and reflects the health status of plants [80]. In Virginia Mallow plants, with increasing concentration of quercetin applied, an increase in chlorophyll content was also observed. The stimulating effect of exogenous application of quercetin on chlorophyll content was also confirmed in studies by other authors [46]. The average chlorophyll content in the leaves of Virginia Mallow ranged from 22 to 48 relative units [81]. In another study, the average amount was 38 relative units for irrigated crops from medium soil and 39 relative units for rainfed crops from medium and poor soil [56]. The chlorophyll content in leaves is also a useful tool for monitoring plant productivity [82]. The use of an aqueous quercetin solution can stimulate the tolerance of plants to abiotic stresses by enhancing antioxidant enzymes and securing photosynthetic activity, as well as preventing membrane peroxidation or strengthening the plant defence system against oxidative damage.

Chlorophyll fluorescence is a non-invasive measure of photosystem II (PSII) activity and is a widely used technique in plant physiology. The sensitivity of PSII activity to abiotic and biotic factors has made it a key technique, not only for understanding the mechanisms of photosynthesis but also as a broader indicator of how plants respond to environmental changes [83]. This is because photosynthesis is related to all the metabolic and physiological processes that occur in the plant cell, and any change in the environment that causes changes in these processes will affect the photosynthetic process [84]. Nutrient deficiencies and abiotic stresses that occur during the growing season of plants directly affect the photosynthetic apparatus. Usually, a decrease in photosynthetic efficiency is the first symptom of the negative impact of stress on the plant, which can affect the maximum quantum efficiency of PSII and which is proportional to the F_v_/F_m_ ratio, reflecting the efficiency of light in primary photosynthesis [85]. In the experiment, all chlorophyll fluorescence tested parameters showed a significant increase in value compared to the control and with an increase in the applied concentrations and the duration of the experiment. Quercetin plays a role in the repair processes of the photosynthetic apparatus, leading to an increase in photosynthetic efficiency, which was confirmed in studies by other authors, where the chlorophyll fluorescence parameters increased as a result of the external application of quercetin [86,87]. Stimulating plants allows the occurrence of harmful cellular mechanisms. In the case of quercetin, this paradoxical phenomenon is known. Therefore, it is also important to determine the critical value at which the effect of the action will not negatively affect the given plant.

## 4. Materials and Methods

### 4.1. Experimental Design

The Virginia Mallow seeds were seeded in pots with a diameter of 20 cm, in which 5 kg of soil with a grain size composition of slightly acid loamy sand (pH: KCl 6.37; H_2_O 6.50) was placed. The experiments were carried out in four replicates of 10 pots per variant in a growth chamber at a temperature of 22 ± 2 °C, humidity of 60 ± 3% RH, photoperiod of 16/8 h (day/night) and maximum light intensity of approximately 300 µE m(2∙s^−1^). The substrate humidity was maintained at 60% of the field water capacity. Twenty seeds were sown in pots, and after emergence the number of plants was reduced to 6 per pot. The positions of the pots in the experiment were randomly selected each week. On the 30th day after the emergence of Virginia Mallow plants (plant height 8–12 cm), salt stress was applied by watering the soil with 100 mL of 200 mM sodium chloride (NaCl) per pot (salinity light). At the same time, control samples were watered with the same volume of deionized water. On the 40th day after emergence, the plants were sprayed with an aqueous quercetin solution at concentrations of 1.0%, 3.0% and 5.0% in the amount of 10 mL per pot by a hand sprayer. Plants subjected to salt stress (without Q spray) and plants subjected to only quercetin solutions (Q1%, Q3%, Q5%) (without salt stress) were used as a control. A uniform spraying procedure was used: the same amount of solution was used for each pot until the solution was completely exhausted. At the same time, the control sample was filled with deionized water at the same volume. The spray was carried out with a handheld laboratory sprayer with flow regulation with a dose volume of 1.2 mL ± 0.1 during one press (outlet diameter 0.6 mm). Physiological measurements taking place on Virginia Mallow leaves (gas exchange, relative chlorophyll content, and chlorophyll fluorescence) were performed three times: on the 1st (term 1), 7th (term 2) and 14th (term 4) day after spraying treatment.

### 4.2. Measurement of Gas Exchange

The LC pro-SD photosynthesis measurement system (ADC Bioscientific Ltd., Herts, UK) equipped with a flow chamber with a flow accuracy of ±2% of its range was used to measure the gas exchange parameters: photosynthetic network intensity (P_N_), transpiration rate (E), stomatal conductance (g_s_) and intercellular CO_2_ concentration (C_i_). During measurement, the light intensity was 1500 mol m^−2^∙s^−1^ and the temperature in the measurement chamber was 28 °C. Measurement was carried out in 3 replicates per pot near the middle of the leaf blade [88].

### 4.3. Measurement of Chlorophyll Content Index

A CCM 200 meter (Opti-Sciences Inc., Hudson, NH, USA) was used to measure CCI in leaves. Measurements were taken from three fully expanded leaves near the mid-leaf blade. Measurements were taken in 10 replicates per pot [88].

### 4.4. Measurement of Chlorophyll Fluorescence

The maximum quantum yield of photosystem II (PSII) photochemistry (F_v_/F_m_), the maximum quantum yield of primary photochemistry (F_v_/F_0_), and the photosynthetic efficiency index (PI) were measured on three fully expanded leaves. A Pocket PEA continuous excitation fluorometer (Pocket PEA, Hansatech Instruments, Pentney, Great Britain) equipped with black shading clips was used to perform the measurements. The clips were placed near the mid-blade of the leaf (away from the midrib of the leaf). Triplicate measurements were made in each pot after 30 min of dark adaptation. The fluorescence signal was collected under red actinic light with a maximum light source wavelength of 627 nm. This was administered for 1 s at a maximum available intensity of 3500 μmol (photon) of photosynthetically active radiation (PAR) [88].

### 4.5. Statistical Analysis

To verify the normality of the distribution, the Shapiro–Wilk test was performed at α = 0.05. Then the homogeneity of variance was verified. A two-factor ANOVA test with repeated measures (with time as a factor) was used. To determine and verify the relationship, Fisher’s LSD post hoc test was performed at the significance level of α ≤ 0.05. Statistical analysis was performed using Statistica 13.3.0.

## 5. Conclusions

The applied aqueous solution of quercetin had a positive effect on the physiological properties of plants of Virginia Mallow. The stimulating effect of the solution was observed for each of the concentrations analysed. An increase in the values of P_N_, E, g_s_, CCI, F_v_/F_m_, F_v_/F_0_, PI and a decrease in the C_i_ value were observed. At the same time, no deterioration was observed in the condition of the plants. The studies carried out may contribute to an increase in the yield of the above-ground mass of Virginia Mallow plants and thus increase the efficiency of cultivation of this species. Furthermore, these results can be used as a basic study to develop a strategy to reduce the negative impact of abiotic stresses on agriculture productivity, including crops intended for energy purposes. It seems that the foliar application of quercetin can be used as an effective and environmentally friendly way to reduce the impact of soil salinity on crops, but more research is needed on this issue, especially in field conditions.

## Data Availability

The data presented in this study are available from the corresponding author upon reasonable request.
